# Taming Postoperative Delirium with Dexmedetomidine: A Review of the Therapeutic Agent’s Neuroprotective Effects following Surgery

**DOI:** 10.3390/ph16101453

**Published:** 2023-10-13

**Authors:** Vincent Bargnes, Brian Oliver, Emily Wang, Seth Greenspan, Zhaosheng Jin, Isaac Yeung, Sergio Bergese

**Affiliations:** 1 Department of Anesthesiology, Stony Brook University Hospital, Stony Brook, NY 11794, USA; 2 Renaissance School of Medicine, Stony Brook University, Stony Brook, NY 11794, USA

**Keywords:** postoperative delirium, dexmedetomidine, perioperative neurocognitive disorder, neuroprotection

## Abstract

Postoperative delirium (POD) represents a perioperative neurocognitive disorder that has dreaded ramifications on a patient’s recovery from surgery. Dexmedetomidine displays multiple mechanisms of neuroprotection to assist in preventing POD as a part of a comprehensive anesthetic care plan. This review will cover dexmedetomidine’s pharmacological overlap with the current etiological theories behind POD along with pre-clinical and clinical studies on POD prevention with dexmedetomidine. While the body of evidence surrounding the use of dexmedetomidine for POD prevention still requires further development, promising evidence exists for the use of dexmedetomidine in select dosing and circumstances to enhance recovery from surgery.

## 1. Introduction

The perioperative setting is host to a variety of potential neurocognitive insults. Even when patients are optimized for elective surgery they are often famished, stressed, and in a state of discomfort. From the moment they enter the preoperative staging area till discharge, patients are in an unfamiliar and institutional environment that regularly interrupts them and imposes limitations on visitation. During the perioperative period, patients are exposed to various stressors, including surgical trauma, anesthesia, pain, disturbances to sleep, and changes in nutrition. These and other factors contribute to the prevalence of postoperative neurocognitive complications, especially in patients who are at high risk.

One of the responsibilities of an anesthesiologist is to minimize the stresses patients face before, during, and after surgery. Fortunately, modern advancements in the field of anesthesiology and perioperative medicine equip anesthesiologists with an armamentarium of techniques to help protect their patients, including the focus of this article—dexmedetomidine. The Federal Drug Administration has approved dexmedetomidine for the sedation of patients in an intensive care setting who are intubated and mechanically ventilated. The periprocedural sedation of non-intubated patients and the use of dexmedetomidine in patients under 18 years of age is beyond the scope of this review and is covered elsewhere [1]. Dexmedetomidine is a sedative and analgesic, working mainly via selective alpha-adrenergic receptor agonisms with action primarily taking place on the central nervous system. The characteristic neuroprotective properties observed with dexmedetomidine, at least partially through the imidazoline receptor agonism in pre-clinical and clinical investigations, may help dull the neurocognitive affronts patients face perioperatively.

## 2. Perioperative Neurocognitive Disorders

Emergence delirium, postoperative delirium (POD), and postoperative cognitive dysfunction (POCD) are the three major perioperative neurocognitive disorders. Each represents changes in mental status and cognition with varying timeframes. Emergence delirium occurs during or immediately after emergence from anesthesia, compared to postoperative delirium (POD) which has a delayed onset between postoperative days 1 and 5 [2,3]. POCD represents a more chronic disorder that is centered on the decline of a patient’s cognitive ability without criteria established by the most recent edition of the Diagnostic and Statistical Manual of Mental Disorders (DSM) [4]. Some authors believe that POCD is detectable as early as 7 days after surgery with effects lasting 1 year or more [5,6]. These three disorders may represent a spectrum of perioperative changes with distinct impacts on a patient’s life; however, this article will focus primarily on POD.

While the most recent edition of DSM does not specifically define POD, the manual lists five criteria for the diagnosis of delirium. Delirium is defined as a disturbance in attention, awareness, and cognition from baseline that develops over a short period of time and tends to vary in severity over the course of the day. Evidence is required to prove that the delirium episode is secondary to a physiological consequence of a medical condition, substance use, toxin exposure, or multiple factors [4].

Because POD is not uniformly defined, there is heterogeneity amongst trials and scientific reviews focused on POD. However, clinical assessment tools have been developed to assist in recognizing delirium, including the validated Confusion Assessment Method (CAM) and Delirium Rating Scale-Revised-98 (DRS-R-98) [7,8]. These were developed as the DSM began to define delirium in the third and fourth editions, released in 1980 and 1994, respectively.

### 2.1. Postoperative Delirium Risk Factors

While the risk of POD is 2–5% of all patients undergoing surgery, the risk is multiplied ten-fold or more in patients with risk factors, including age greater than 60 years and a pre-existing diagnosis of dementia, cardiovascular disease, diabetes mellitus, or anemia [9,10,11,12,13,14]. Cognitive decline prior to surgery, defined as significant reductions relative to a patient’s baseline cognitive level, has also been shown as a risk factor in developing POD [15]. In addition to risk factors associated with patient comorbidities, the type of surgery can also play a role in increasing POD incidence. Emergency surgery and complex surgeries, including elective cardiac surgery, liver transplant, and pulmonary endarterectomy, carry an elevated risk of POD with incidence up to 56% [16,17,18,19,20]. Whenever possible, all patients with risk factors for POD should be identified early on in their perioperative course and their anesthesia care plan should consider preventing POD interventions that are discussed later in this article, including but not limited to dexmedetomidine.

Perioperative pharmaceuticals can also be risk factors for POD. In 2016, a study revealed that preoperative delirium-inducing medications, including high-dose narcotics, benzodiazepines, and anticholinergics were associated with the development of POD following oncologic surgery [21]. More recent trials have further investigated the individual effects of the medications. Long-term preoperative use of anticholinergic medication in patients 65 years of age or older undergoing oncologic surgery was found to be associated with POD, with stronger anticholinergic medications associated with a higher risk of POD [22]. A study of adults aged 70 years or older without dementia undergoing major elective non-cardiac surgery evaluated perioperative benzodiazepines, beta-blockers, non-steroid anti-inflammatory drugs (NSAIDs), opioids, and statins on POD and found that only postoperative benzodiazepine administration was associated with POD [23]. Further research is required to elaborate on which medications and administration timeframes should be avoided to minimize POD.

### 2.2. Postoperative Delirium Sequalae

POD drastically impacts a patient’s recovery and postoperative outcomes. A 2022 investigation that studied patients 70 years old and older undergoing non-emergent cardiac and orthopedic surgery revealed that the presence of POD, as evidenced by one of four validated assessment tools, between postoperative days 1 through 5 increased the total length of hospital stay by 8 days and increased time in the intensive care unit (ICU) by 6 days [24]. Extended hospital and ICU length of stay due to delirium has also been seen amongst patients as young as 50 years old and seen in other surgical disciplines, including gastrointestinal surgery [25].

Patients who experience POD are at high risk of functional decline after discharge and often require discharge to care facilities. POD following surgery for colorectal cancer was associated with loss of independence as measured through activities of daily living (ADL). A subpopulation analysis of patients 80 years old or older revealed an association between POD and loss of independence at 30 days post-discharge [26]. The prior literature on patients 70 years of age or older undergoing elective major orthopedic, vascular, or abdominal surgeries corroborates this reduction in independence with a 50% increased risk of discharge to a nursing home, subacute rehabilitation facility, or acute rehabilitation facility following POD [27]. Even when patients who suffered POD in the setting of elective surgery for colorectal cancer or abdominal aortic aneurism repair and regained their physical health status, they were more likely to experience a decline in their quality of life including their psychological health, social relationships, and environment at one year post-surgery [28].

These sequelae come with remarkable healthcare costs, making the mitigation of POD a prime target to curb Medicare spending. A study of patients 70 years of age or older undergoing elective surgery investigated the healthcare costs of patients who developed POD compared to those who did not. Major costs included readmissions and institutional rehabilitation stays. The incidence of delirium resulted in USD 44,291 in additional costs per patient per year with a direct increase in cost to USD 55,474 when the patient’s CAM score indicated severe delirium. The authors extrapolated their findings and found that USD 32.9 billion is spent in the United States on POD sequelae costs per year [29]. A single intervention should not be expected to eliminate POD’s nationwide economic impact, but a nationwide reduction in POD by 4% has the potential for over USD one billion in cost savings.

Healthcare costs aside, patients with POD also suffer from an increased rate of mortality. Amongst patients who underwent bowel resection, those with POD experienced a 14% hospital mortality rate compared to a 0.1% mortality rate in those who did not develop POD [16]. This mortality disparity was replicated again in patients undergoing complicated type B aortic dissection repair with a significant increase in mortality at long-term follow-up. At 5 years post-operation, patients with POD had a 58% survival rate, while those without POD experienced a near doubling of their survival rate at 97% [30].

The sequelae of POD should be made clear to all patients at high risk of POD prior to surgery, as well as to their caregivers. An ideal setting for this is a dedicated preoperative clinic with thorough preoperative assessment, counseling, and optimization that includes interprofessional collaboration with surgical team members [31,32]. Obtaining informed consent for anesthesia includes discussing the risks associated with anesthesia, such as POD, and can also be used to launch a discussion to help build the highest quality anesthesia care plan for the patient, which may include POD prevention strategies.

## 3. Etiology of Postoperative Delirium

**Figure 1:**
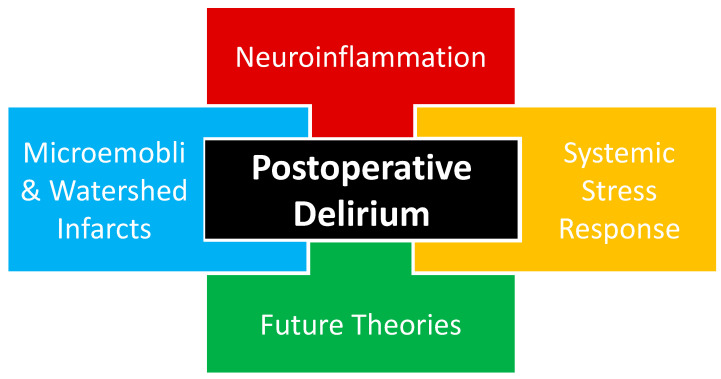
Current proposed etiological theories of postoperative delirium likely interact in a multifactorial fashion, including neuroinflammation, systemic stress response, and cerebrovascular events. An additional etiologic component of postoperative delirium is the theories that will emerge based on future investigations that may help us to better understand the etiology of postoperative delirium.

The complete pathophysiology of POD remains unknown with multiple theories proposed, including neuroinflammation, systemic stress response, and microemboli or watershed stroke. POD is likely multifactorial in nature, as a combination of proposed and yet-to-be-proposed etiologies that will be presented in future works (Figure 1).

### 3.1. Neuroinflammation

Evidence of inflammation has been associated with POD through studies correlating inflammatory biomarkers and POD assessment tools, implicating neuroinflammation in POD (Figure 1). A meta-analysis of a variety of surgeries from cardiac to abdominal to orthopedic found a relationship between POD and both non-specific inflammatory biomarkers, interleukin-6 (IL-6) and c-reactive protein (CRP), and neuronal injury biomarkers, neurofilament light chain (NfL) and S100 calcium-binding protein B (S-100B) [33]. Tau, a microtubule-associated protein, also serves as a neuronal injury biomarker associated with the POD. The magnitude of tau elevation corresponded with POD severity and decreased as POD features improved in a prospective cohort study evaluating patients undergoing elective non-cardiac and non-neurosurgical operations [34].

Blood–brain barrier damage can be a sequela of neuroinflammation and has been associated with POD following non-intracranial surgery using the blood–brain barrier damage surrogates cerebrospinal fluid/plasma albumin ratio and plasma S100B. Elevated levels of these blood–brain barrier damage surrogates are also correlated with POD severity [35].

The neutrophil-to-lymphocyte ratio (NLR) is a white blood cell-derived inflammatory biomarker readily available in many patients perioperatively and is associated with POD. An investigation into lower limb fracture surgery found that a postoperative NLR of 10.2 or above was strongly associated with POD [36]. The same study found that a POD prediction model that took into consideration multiple factors, including age, perioperative benzodiazepine administration, change in CRP, and change in white blood cell-derived biomarkers, was able to better predict POD than a change in NLR alone. In addition to finding utility in a clinically ubiquitous inflammation marker, this finding supports POD having a multifactorial etiological basis.

### 3.2. Systemic Stress Response

Surgical trauma triggers a stress response that may also be implicated in the pathophysiology of POD. Surgery activates the hypothalamic–pituitary–adrenal (HPA) axis and causes substantial neuroendocrine changes designed to facilitate the body’s response to trauma. Like accidental trauma, surgical trauma is sensed by the hypothalamus through changes in hemodynamics and inflammatory markers. These changes initiate a potent HPA cascade with effects on multiple organ systems and hormones, including the posterior pituitary, anti-diuretic hormone, anterior pituitary, adrenocorticotropic hormone, growth hormone, adrenal cortex, cortisol, adrenal medulla, catecholamines, arteriolar smooth muscle, renal blood flow, renin, angiotensin, aldosterone, pancreas, glucagon, and insulin (Figure 1) [37]. A multitude of factors can dysregulate the HPA axis, such as age, deconditioning, and renal disease, and therefore disrupt this intricate coordination with an inadequate or overexaggerated reaction [38]. A meta-analysis revealed that patients above 60 years of age have higher cortisol levels postoperatively compared to their younger peers [39]. While this may correspond to a higher baseline cortisol level as humans age, elevated postoperative cortisol levels have been associated with POD [40]. Cortisol’s wide-reaching impacts on the body, including psychogenic effects, must be taken into consideration when considering the etiology of POD.

### 3.3. Microemboli or Watershed Stroke

Cerebrovascular incidents have also been proposed as a theory in the development of POD, with the disruption of reliable perfusion to the brain thought to contribute to a patient’s altered mental status after surgery. Clamping and manipulation of the aorta during off-pump coronary artery bypass grafting (CABG) surgery has been shown to cause multiple cerebrovascular embolic events seen on a transcranial Doppler [41]. The anaortic method of off-pump CABG features no aorta clamping or manipulation and has been associated with a three-fold reduction in POD [42]. These findings both support the microemboli theory of POD and provide a POD mitigation strategy for patients undergoing surgery involving aorta manipulation.

Sections of the brain located between two arterial supplies are classified as border zones or watershed areas and rely on uninterrupted perfusion, especially in advanced cerebrovascular disease. During times of reduced cerebral blood flow, there is a risk for watershed infarction, resulting in damage to a considerable amount of the brain. Peripheral arterial disease (PAD) has been associated with underlying cerebrovascular disease [43]. Patients with PAD undergoing elective lower extremity bypass surgery were found to have a greater incidence of POD with more advanced PAD, measured by both lower ankle-brachial index values and higher Rutherford class [44]. The above vascular and cardiothoracic surgery investigations have both demonstrated that a disturbance in cerebrovascular perfusion has been associated with POD and lend credence to a microemboli or watershed stroke theory being involved with the etiology of POD (Figure 1).

## 4. Dexmedetomidine

### 4.1. Alpha-Adrengeric Agonism

**Figure 2:**
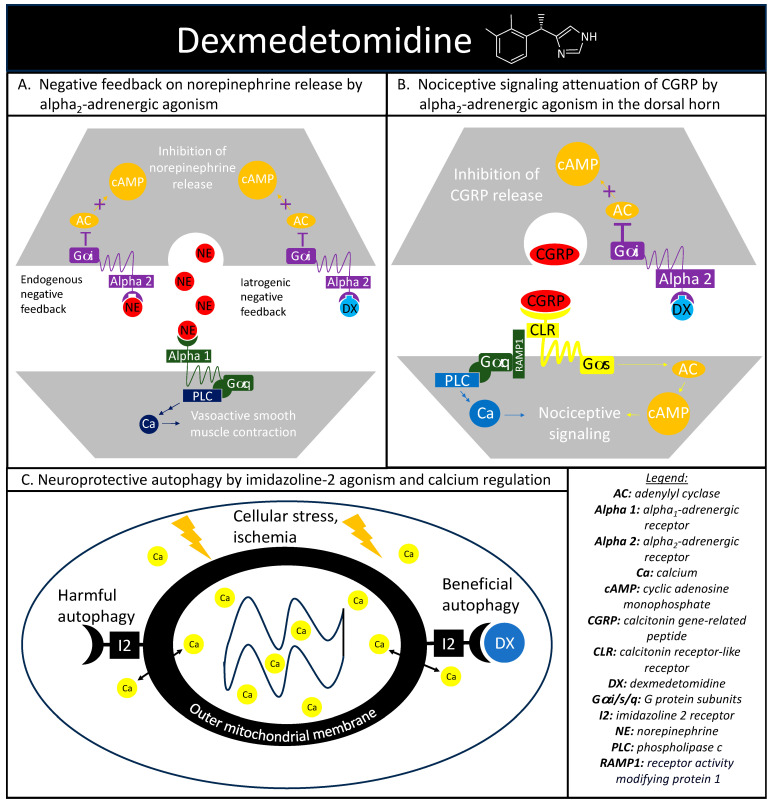
Dexmedetomidine’s highlighted mechanisms of action. (**A**) depicts the cellular signaling pathways present during the binding of norepinephrine to alpha_1_- and alpha_2_-adrenergic receptors. Alpha_2_-adrenergic receptors play a role in an endogenous negative feedback loop to reduce norepinephrine release, which is the mechanism that dexmedetomidine is designed to mimic. (**B**) depicts dexmedetomidine’s cellular signaling pathway behind the therapeutic agent’s antinociception through alpha_2_-adrenergic activation in reducing the release of calcitonin gene-related peptide and subsequent nociceptive signaling. (**C**) depicts the basic interaction between dexmedetomidine and imidazoline-2 receptors on the outer mitochondrial membrane in regulating intracellular calcium levels and producing beneficial autophagy in the setting of cellular stress.

The alpha-adrenergic receptors play a vital role in maintaining proper hemodynamics and play a substantial role in the stress response previously discussed. Norepinephrine is a catecholamine released during a stress response with vasoconstrictive effects mediated through the alpha_1_-adrenergic receptor agonism, with alpha_2_-androgenic receptors functioning as a negative norepinephrine feedback loop on the presynaptic nerve terminal [45]. Dexmedetomidine is a sedative that exploits this intrinsic negative feedback loop through selective alpha_2_-adrenergic receptor agonisms (Figure 2A). Activated alpha_2_-andregneic receptors attenuate noradrenergic pathways through the decrease in norepinephrine release from the locus ceruleus through an inhibitory G-protein coupled receptor action causing a reduction in adenylyl cyclase activity and cyclic adenosine monophosphate levels. The alpha_2_-adrenergic receptors also explain the analgesic effect seen with dexmedetomidine. Within the dorsal horn of the spinal column, there are also alpha_2_-adrenergic receptors and the agonism of these receptors reduces the release of substance P and calcitonin gene-related peptide (CGRP) and mitigates nociceptive signaling (Figure 2B) [46].

Clonidine, an anti-hypertensive medication, is similar to dexmedetomidine as they both exhibit alpha-adrenergic agonisms. However, dexmedetomidine is nearly eight times more selective for alpha_2_-adrenergic receptors with more sedation, analgesia, and hemodynamic stability. There is an expected level of hemodynamic change, including hypotension and bradycardia, when administering dexmedetomidine partially due to the dampening of the adrenergic system but also the imperfect selectivity of alpha-adrenergic receptors. The selectivity for alpha_2_-adrenergic receptors is minimized at high doses or with rapid intravenous administration [45]. To minimize adverse effects from high doses or rapid administration, dexmedetomidine should be introduced over a prolonged bolus, around 10 min, and should be titrated carefully to a patient’s individual hemodynamic measures, especially heart rate and blood pressure [47].

### 4.2. Imidazoline Agonism

Dexmedetomidine also has an affinity to receptors outside of the alpha-adrenergic system, including imidazoline type 2 (I2) receptors. While there is a paucity of research surrounding dexmedetomidine and I2 receptors, evidence suggests that dexmedetomidine-I2 binding can regulate calcium levels within chromaffin cells and may be protective against neuronal damage [48]. Additional evidence has implicated the I2 receptor agonism in the regulation of cytosolic calcium concentrations at the level of the outer mitochondria membrane and in the mitigation of endoplasmic reticulum stress-induced apoptosis (Figure 2C) [49].

### 4.3. Neurotransmitter Alteration

Dexmedetomidine may exert its neuroprotection via the alteration of neurotransmitter pathways including catecholamines, glutamate, acetylcholine, and butyrylcholine. A pre-clinical trial explored the effects of dexmedetomidine on cerebral neurotransmitter concentrations in rats with induced cerebral ischemia. The animals were assigned to three groups including one control group, one experimental group receiving 100 mcg/kg of intraperitoneal dexmedetomidine 30 min prior to ischemia, and one group of sham-operated rats. Concentrations of cerebral catecholamines and glutamate, as well as plasma catecholamines, were measured, with a resulting 95% reduction in circulating plasma norepinephrine in rats pre-treated with dexmedetomidine. However, cerebral concentrations of both norepinephrine and glutamate remained increased in response to ischemia despite treatment with dexmedetomidine. The results of this study suggest that dexmedetomidine’s neuroprotective effects are independent of presynaptic norepinephrine or glutamate release in the brain [50].

Another recent study examined the effects of dexmedetomidine on cholinergic dysregulation as well as the altered inflammatory response to surgical trauma known to be associated with POD. Two enzymes at play here are acetylcholinesterase (AChE) and butyrylcholinesterase (BChE). These researchers performed a secondary analysis of a randomized, double-blinded, placebo-controlled trial which had demonstrated a lower incidence of POD in patients given dexmedetomidine. In this analysis, cholinesterase levels were measured preoperatively and twice postoperatively, showing no change in ache activity and an initial decrease in B.Ch.E. activity followed by rapid recovery in those treated with dexmedetomidine. This is compared to the significant decrease in both enzymes in the placebo group. The results of this study may point towards the alteration of the cholinergic anti-inflammatory pathway as the mechanism for dexmedetomidine’s neuroprotective effects and decreased rates of POD [51].

Although further research is needed to explore the exact mechanism of neuroprotection from dexmedetomidine, these studies demonstrated a link between dexmedetomidine and the alteration of catecholamine levels. Lower circulating plasma norepinephrine and a limited decrease in AChE and BChE after surgery may result in less inflammation and a less robust systemic response to surgical stress, resulting in neuroprotection.

### 4.4. Pharmacodynamics and Drug–Drug Interactions

When given as a loading dose intravenously, dexmedetomidine exhibits a rapid onset of action in 5 to 10 min with peak effect seen in 15 to 30 min. This onset of action is prolonged to 60 min when given as a continuous intravenous infusion. The highly lipophilic nature of dexmedetomidine facilitates rapid distribution with a 6 min distribution half-life, producing rapid onset. The duration of effect is dependent on administration, given the notable context-sensitive half-time, over 4 h after an 8 h infusion.

The potentiation of other pharmaceuticals has been observed with dexmedetomidine administration, including reducing benzodiazepine requirements and opioid-sparing effects [52,53]. Therefore, the effects of benzodiazepines and opioids may be greater in a patient undergoing treatment with dexmedetomidine than without co-administration of dexmedetomidine. Dexmedetomidine is metabolized by the liver via direct glucuronidation and CYP2A6 metabolism followed by clearance in the urine and secondarily in feces [54,55]. Dose adjustments must be considered in patients with liver function impairment and those taking medications that manipulate the activity of CYP2A6, such as amiodarone and phenobarbital.

### 4.5. Pre-Clinical Data

Immune cells within the central nervous system are at least partially responsible for neuroinflammation, especially microglia, the central nervous system-specific and primary immune cell. In a pre-clinical study of human microglial cells, cells were stress-stimulated through exposure to lipopolysaccharide (LPS), a component of gram-negative bacterial cell walls, leading to an increase in an inflammatory marker also associated with POD, IL-6. While the investigation was not able to significantly reduce levels of IL-6 when stressed by LPS, unstressed cells exposed to dexmedetomidine had significantly decreased production of IL-6 compared to unstressed cells not exposed to dexmedetomidine [56]. This demonstrates that dexmedetomidine does hinder the body from mounting an appropriate response to infection but may support the role of reducing neuroinflammation when exposed to a different type or level of stress.

In a similar trial, rats were exposed to ischemia-reperfusion injury followed by either dexmedetomidine or saline intravenously with levels of neuronal injury biomarkers, including S100B, obtained after injury. In the group with injury who were then treated with dexmedetomidine, there was a significant reduction in S100B compared to the group with injury who were then treated with saline [57]. Additionally, there was a significant increase in superoxide dismutase, an agent that intercepts free radicals and spares tissues experiencing damage, among rats that were exposed to dexmedetomidine with a corresponding reduction in damage to the hippocampal CA1 region, an area key to memory and consciousness. This trial supports the role of dexmedetomidine in improving POD in the setting of both cerebral perfusion disruption and neuroinflammation.

This preservation of hippocampal architecture was further elaborated by a pre-clinical trial that exposed rats to abdominal surgery with ischemia of the superior mesenteric arterial supply. Rats were divided into pre-treatment with either dexmedetomidine or saline before surgery. In the dexmedetomidine pre-treatment group, there was a significant decrease in inflammatory markers, including IL-6 [58]. When the dexmedetomidine pre-treated group was exposed to yohimbine, an alpha_2_-adrenergic receptor antagonist, there was no significant change compared to the saline pre-treatment group, implying that this reduction in inflammatory markers is mediated by the alpha_2_-adrenergic receptor agonism of dexmedetomidine.

Again, there was visibly reduced nerve cell loss on hippocampal tissue sampling when they were pre-treated with dexmedetomidine [58]. This group of investigators had two groups pre-treated with dexmedetomidine, one that was exposed to 3-methyladenine, an autophagy inhibitor, and another that was able to undergo autophagy. The group that had autophagy inhibited did not have a reduction in nerve cell loss on tissue sampling, implying that dexmedetomidine’s hippocampal safeguarding effect may be due to beneficial autophagy (Figure 2C). While no imidazoline receptor antagonist was utilized in this study, there is an overlap between control of intracellular calcium, I2 receptors, and autophagy.

### 4.6. Clinical Data Risks, Benefits, and Alternatives

The above basic science investigations are supported by a meta-analysis of clinical trials investigating dexmedetomidine in the prevention of a perioperative neurocognitive disorder following a variety of surgical procedures, including liver transplant, prostatectomy, femoral head replacement, and CABG. Mirroring the pre-clinical data, the use of dexmedetomidine was associated with a significantly reduced level of the inflammatory marker IL-6 and a neuronal injury biomarker, neuron-specific enolase (NSE) [59]. Additionally, the incidence of perioperative cognitive disorders was reduced within the dexmedetomidine group.

A randomized clinical trial investigated the effects of dexmedetomidine on POD and inflammatory markers in patients 65 years or older undergoing acute hip fracture surgery and further validated the above pre-clinical and clinical findings. The incidence of POD was halved, 26% compared to 13%, when dexmedetomidine was used perioperatively [60]. IL-6 and another inflammatory marker, tumor necrosis factor-alpha, were significantly reduced postoperatively. Tachycardia, bradycardia, hypertension, and hypotension were all monitored throughout and showed no difference between the two groups.

Another recent randomized trial exploring the role of intraoperative dexmedetomidine in a patient’s sleep, pain, and delirium after intracerebral resection also paralleled the above results. The incidence of POD was significantly reduced by more than half in patients treated with dexmedetomidine, 22% versus 56% [61]. The patients who received a placebo required more postoperative analgesia than their dexmedetomidine peers. Patients in the dexmedetomidine treatment group also reported better sleep through postoperative day 3. However, this study did detect that the heart rate was significantly lower in the dexmedetomidine group in the final portion of the surgery. Despite this statistically significant decrease in heart rate, there was no difference in mean arterial pressure. This study showed multiple postoperative benefits of dexmedetomidine without clinically significant hemodynamic changes. Allowing for proper sleep is thought to minimize delirium, and this study may represent a signal that beneficial sleep from dexmedetomidine is a part of the logic for reducing the incidence of POD [62]. Frequent blood pressure and heart monitoring is a part of the standard monitoring guidelines recommended by the American Society of Anesthesiologists with these vital signs repeated a minimum of every five minutes while a patient is undergoing anesthesia. This is followed by continual re-evaluation of the patient in the post-anesthesia care unit as the patient recovers from anesthesia. Frequent monitoring allows for the identification of an opportunity to administer medications that can correct changes in blood pressure and heart rate.

In addition to the adverse effects of bradycardia and hypotension, delayed emergence has also been reported with dexmedetomidine. When dexmedetomidine was co-administered with propofol, a randomized prospective trial revealed a significantly longer time to eye opening by an average of 10 min following cessation of anesthesia compared to propofol alone [63]. The study found that when sevoflurane was co-administered with dexmedetomidine, there was no difference in emergence timing. This study highlights an additional side effect to consider while administering dexmedetomidine, especially in the setting of total intravenous anesthesia (TIVA).

Providing patients with alternatives to any intervention is fundamental to informed treatment. This article views dexmedetomidine as a complementary measure of a comprehensive, personalized anesthetic care plan with the aim of preventing POD. Alternatives to dexmedetomidine’s complementary function include other complementary POD prevention measures, such as other pharmaceutical agents and non-pharmaceutical interventions and the absence of dexmedetomidine usage.

Olanzapine is an atypical antipsychotic that has been shown to be an alternative complementary pharmaceutical agent to dexmedetomidine for POD prophylaxis, with POD seen in 14.3% of patients treated with olanzapine compared to 40.2% treated with placebo [64]. Like other pharmaceutical agents, olanzapine carries a risk of adverse effects, including extrapyramidal symptoms. The maintenance of select physiologic variables during surgery, including blood pressure, blood glucose levels, and processed electroencephalography, is associated with a reduction in POD and often requires pharmaceutical intervention. Maintaining hemodynamic stability throughout the case, defined as preventing the mean atrial pressure from falling below 55 mmHg, has been shown to reduce POD [65]. Also, maintaining blood glucose levels between 60 and 150 mg/dL has been shown to prevent POD [66,67]. The use of processed electroencephalography with the bispectral index (BIS) is an additional monitor beyond the standard of care monitoring that can assist in guiding anesthetic depth and has been shown to reduce POD under certain settings, including patients under general anesthesia and spinal anesthesia with sedation [68,69]. However, a more recent study did not find the same POD prevention signal [70].

The evidenced-based Hospital Elder Life Program (HELP) is a postoperative, multicomponent delirium prevention program that addresses risk factors such as sleep–wake cycles, immobility, hydration status, and sensory impairment with POD reductions of up to 40% [71,72]. This multidisciplinary program requires specialized institutional infrastructure for proper execution.

A patient’s specific perioperative circumstances will dictate which POD prevention alternatives are appropriate and available, but complementary measures can be used in combination and may complement each other to enhance perioperative outcomes. Consider a patient who is at high risk for POD, they may be served best by receiving dexmedetomidine perioperatively, undergoing BIS-guided anesthesia, and transferring postoperatively to a HELP. Unfortunately, there is a paucity of data surrounding the use of combining POD prevention therapies at the time of writing. The authors hope that additional evidence will be produced on this topic.

## 5. Clinical Evidence of Postoperative Delirium Prevention with Dexmedetomidine

The effects of dexmedetomidine on perioperative neurocognitive disorders can be broken down based on three categories including non-cardiac and cardiac surgical patient populations, dosing and timing of dexmedetomidine administration, and lastly dexmedetomidine specifically in the ICU population.

### 5.1. Cardiac and Non-Cardiac Surgery

In the non-cardiac adult surgical population, several trials and meta-analyses have shown a decrease in perioperative neurocognitive disorders after administration of dexmedetomidine. In pooled data from thirteen studies, a 40% reduction in PCOD was estimated [73]. In another meta-analysis of 18 RCTs with 3309 patients, dexmedetomidine was associated with a significant reduction in POD (OR 0.35, *p*-value < 0.01). Subgroup analysis of cardiac (nine studies, 1301 patients) and non-cardiac (nine studies, 2008 patients) patients showed a significant difference in POD incidence in both groups favoring dexmedetomidine over control (OR 0.41, *p*-value < 0.01) for cardiac surgery and for non-cardiac surgery (OR 0.33, *p*-value < 0.01). Also demonstrated was a reduced POD incidence in both the elderly age group, defined as 65 years of age or older, as well as the younger group, defined as younger than 65 years of age [74].

Another meta-analysis of 13 randomized controlled trials in 2021 showed that dexmedetomidine administration, either solely intraoperatively or both intraoperatively and postoperatively, significantly decreased POD incidence in patients 65 years of age or older following non-cardiac surgery [75]. It is noteworthy that an increased risk of hemodynamic instability, including bradycardia and hypotension, was associated with perioperative dexmedetomidine administration in this meta-analysis.

In the cardiac adult surgical population, meta-analyses have also highlighted many randomized controlled trials showing a decreased incidence of perioperative neurocognitive disorders. In a 2016 metanalysis of 14 studies and 1702 adult cardiac surgery patients, the perioperative use of dexmedetomidine led to a significant risk reduction (risk ratio 0.35, *p*-value 0.0004) as well as a reduced risk of postoperative ventricular tachycardia (RR 0.28, *p*-value = 0.0002) [76]. These studies did however demonstrate an increased risk of bradycardia (RR 2.23, *p*-value = 0.001). It is worth noting that perioperative dexmedetomidine also seemed to be associated with a decreased risk of atrial fibrillation, a shorter ICU and hospital length of stay (LOS), as well as an increased risk of hypotension, although there was limited evidence to support these findings.

A retrospective study of 1134 patients receiving dexmedetomidine between initiation of cardiopulmonary bypass and the first 24 h postoperatively in the ICU demonstrated reduced POD (5.46% vs. 7.42%, *p*-value = 0.0030) as well as improved in-hospital, 30-day, and 1-year survival rates. These researchers proposed the decrease in mortality seen was attributed to the sympathetic nervous system-stabilizing effects of dexmedetomidine which led to less inflammation as well as reduced impacts of ischemia and reperfusion injury [77].

A large meta-analysis of 14 randomized control trials with 14,139 patients in 2018 analyzed studies where any pharmacologic prevention of POD after on-pump cardiac surgery showed significantly lower rates and durations of POD. Sub-analysis of four of these studies, which included dexmedetomidine administration, revealed a significantly lower duration of POD compared to the control (RR = −1.63, *p*-value < 0.00001) [78].

One last meta-analysis worth noting is a 2021 review covering 24 studies including 3610 total patients comparing the incidence of POD between a perioperative dexmedetomidine group and a control group. The results of this analysis revealed a significantly reduced incidence of POD in the dexmedetomidine group compared to the control group (OR: 0.59, *p*-value = 0.001). Subgroup analysis of patients specifically undergoing CABG also confirmed decreased POD (OR: 0.45, *p*-value = 0.005) as well as in mixed cardiac surgery (OR: 0.68, *p*-value = 0.039). However, no difference in rates of longer-lasting POCD was observed [79].

### 5.2. Intraoperative Dosing Regimen

Although the literature specifically pertaining to the efficacy of specific dosing regimens is currently limited, one recent randomized double-blind controlled trial studied 150 elderly patients undergoing hip replacement under general anesthesia and showed encouraging results in reducing rates of POD and emergence delirium. The patients were randomized to groups based on different dexmedetomidine loading dosages (0.25, 0.5, and 0.75 µg/kg for 15 min) followed by 0.5 µg/kg/h continuous infusion until the completion of the surgery. While all dosage groups were associated with significantly lower POD, the higher-dose groups experienced improved agitation scores compared to the lower-dose group. This significantly improved agitation scoring and was also accompanied by bradycardia and hypotension with higher infusion rates compared to the lower infusion rate group [80]. These results should encourage caution in high-dose perioperative dexmedetomidine among patients 65 years of age or older, especially those with low preoperative blood pressures or heart rates. Certain comorbidities and treatments, such as heart block and beta blockers for the treatment of coronary artery disease, may predispose patients to low preoperative hemodynamic measurements. Beyond hemodynamic considerations, surgical intervention must also be considered. If a perioperative neurologic exam is a surgical requirement, dexmedetomidine has been shown to delay the return to baseline neurologic and cognitive status up to 45 min post-transfusion and should be avoided [81].

Reflection on the previously discussed literature can also provide insights into dosing recommendations. For example, dexmedetomidine was associated with decreased POD as an adjuvant intravenous agent to patients undergoing hip fracture surgery at a rate of 0.5 mcg/kg/h starting 30 min before the start of anesthesia, either spinal or general anesthesia with propofol and sevoflurane for maintenance, and then decreased to a rate of 0.3 mcg/kg/h during the operation. Thirty minutes before the surgery’s end, dexmedetomidine was discontinued without a significant difference in adverse effects between dexmedetomidine and placebo treatment [60]. A variation in dosing was utilized successfully to reduce POD in patients undergoing brain tumor resection with a loading dose of 0.6 mcg/kg followed by continuous infusion at 0.4 mcg/kg/h until closure of the dura. These data suggest that either an infusion that began preoperatively (0.5 mcg/kg/h 30 min prior to induction) or a bolus at the start of the case (0.6 mcg/kg over 10 min) followed by a low dose infusion of 0.3–0.4 mcg/kg/h during the procedure produces the necessary therapeutic level of dexmedetomidine to see protective effects against POD.

Learning from trial dosing that did not detect a reduction in POD with dexmedetomidine administration also provides insight. A randomized clinical trial that provided patients undergoing cardiac surgery with 0.5 mcg/kg/h of dexmedetomidine on the patient’s arrival to the operating room until two hours postoperatively did not reduce POD compared to placebo [82]. While these dosing examples were utilized in different surgery types, successful reduction in POD was only seen with either dexmedetomidine pre-treatment or a loading dose. This concept was supported in a trial of elderly patients undergoing major laparoscopic non-cardiac surgery randomized to dexmedetomidine bolus (1 mcg/kg) followed by continuous infusion (0.2–0.7 mcg/kg/h) till the surgery’s stop time, dexmedetomidine bolus (1 mcg/kg) 15 min before the surgery stop, or saline bolus 15 min before the surgery stop. The results revealed that the intraoperative bolus with continuous infusion was superior to a dexmedetomidine or saline bolus near emergence in preventing POD. However, a near-emergence bolus of dexmedetomidine is more effective than a placebo at reducing the duration of delirium. With the differences present in the investigation referenced above, further studies are required to produce robust, evidence-based dosing guidelines for all patients.

The most recent professional society guideline is the 2020 American Society for Enhanced Recovery (ASER) and Perioperative Quality Initiative (POQI) Joint Consensus Statement, which reviewed six studies that used dexmedetomidine for prophylactic POD prevention, including two with patient populations already admitted to an ICU. Based on the available evidence three years ago, they found that the use of dexmedetomidine for POD prevention had mixed results with benefits seen in select patient populations and administration methods. Ultimately, the joint statement reported that there was insufficient evidence to recommend POD prophylaxis with medications [32]. With additional studies now available, future iterations of professional guidelines may update their recommendations to reflect recent results to favor interventions, such as properly timed and dosed dexmedetomidine.

### 5.3. Postoperative Infusion in the Intensive Care Unit

Dexmedetomidine infusions are frequently used in ICU patients for sedation and the prevention of agitation and delirium. One meta-analysis focused on dexmedetomidine infusion compared to other pharmacological interventions in ICU patients, showed a reduction in the incidence of POD. Dexmedetomidine was compared to lorazepam, midazolam, and propofol, and showed decreased LOS in the ICU, the duration of mechanical ventilation, and the incidence of POD (RR = 0.812, *p*-value = 0.020). Dexmedetomidine was also shown to be associated with increased incidences of bradycardia (RR = 1.947, *p* = 0.001) and hypotension (RR = 1.264, *p* = 0.038) [83].

The role of surgical stress in neuroinflammation and delirium was highlighted by a randomized controlled trial of patients being admitted to the ICU status post non-cardiac surgery which revealed a 60% decrease in the occurrence of POD following the administration of dexmedetomidine [84]. Long-term follow-up data showed that survivors at the 3-year mark experienced enhanced cognitive function and a better quality of life. This study also observed an increase in survival rates up to 2 years after low-dose dexmedetomidine infusion in non-cardiac surgical cases [85].

A 2017 meta-analysis of eight randomized clinical trials with 969 cardiac surgery patients who were postoperatively sedated with either dexmedetomidine or propofol demonstrated a decreased risk of POD in the dexmedetomidine group (0.40 RR, *p*-value = 0.0002) and a shorter length of intubation (mean difference −0.95 h, *p*-value < 0.00001). Again, there was a noted increased incidence of bradycardia (RR 3.17, *p*-value = 0.005); however, no difference in the incidence of perioperative hypotension was shown [86].

Unlike the intraoperative pharmaceutical prophylaxis of POD, the 2020 ASER and POQI Joint Consensus Statement strongly recommended that during periods of postoperative mechanical ventilation when patients are sedated, ICUs include dexmedetomidine as a sedative to reduce the risk of POD [32]. This recommendation was based on studies that utilized dexmedetomidine both as a titratable infusion to a specific sedation level with a maximum infusion of 0.7 mcg/kg/h and a constant infusion at a rate of 0.1 mcg/kg/h [84,87].

## 6. Conclusions

Perioperative neurocognitive disorders, specifically POD, represent an area in need of neural protection and repair. The impact of POD and similar disorders on a patient needing surgery can be life-altering. Dexmedetomidine shows repetitive promise across pre-clinical and clinical trials as a sedative, analgesic, neuroprotectant, anxiolytic, and potentiator within a multimodal anesthetic care plan. The mechanisms of action seen in dexmedetomidine administration act as counterpoints to many of the proposed pathophysiologic components of POD and may be a welcome step toward improving a patient’s recovery from surgery. Given that the pathophysiology of POD is still not completely understood, it is anticipated to evolve with future findings and is likely a multifactorial disease; the prevention and treatment of POD require a multifaceted approach that warrants consideration of complementary interventions by anesthesiologists, including dexmedetomidine, close intraoperative monitoring, and discharge from a post-anesthesia care unit to a specialized recovery unit versed in POD.

While the adverse effects observed with dexmedetomidine may or may not be clinically significant, anesthesiologists must balance the risks and benefits of each intervention within a personalized anesthetic care plan for a patient’s surgery. Patients at high risk of perioperative neurocognitive disorders often face multiple comorbidities and require special consideration in their perioperative care plan. Unlike other therapeutic agents used perioperatively, there is not yet a reversal agent for dexmedetomidine on the market for human use.

Future research is needed to optimize dosing recommendations to maximize the therapeutic agent’s benefits while minimizing the adverse effects, especially in vulnerable populations. Knowing the lowest dose across multiple patient populations and surgical characteristics to prevent POD would be a valuable addition to the literature. Additionally, knowing the effect of combining dexmedetomidine with other interventions to prevent POD would be useful in perioperative medicine. At the time of writing, there are active studies available on ClinicalTrials.gov involving the fields of gynecology, neurosurgery, and general surgery to expand the current knowledge base. As the field of anesthesiology progresses and takes care of an older and more medically complex population, anesthesiologists must be on guard against perioperative neurocognitive disorders and act to assist patients with all appropriate measures.

## Data Availability

Data sharing not applicable. No new data were created or analyzed in this study.
